# Missing data handling in pediatric appendectomy research: current practices and National Surgical Quality Improvement Program (NSQIP) analysis

**DOI:** 10.1007/s00464-026-13123-7

**Published:** 2026-07-27

**Authors:** Ayaka Tsutsumi, Tong Si, Joanne Salas, Anna Hargrave, Grace Trello, Jessica Qiu, Priya Reddy, Maria Nash, Nikita Kohli, Shin Miyata

**Affiliations:** 1 Department of Pediatric Surgery, SSM Health Cardinal Glennon Children’s Hospital, 1465 S Grand Blvd, Saint Louis, MO 63104 USA; 2 The Advanced Health Data (AHEAD) Institute, Saint Louis University Hospital, St. Louis, MO USA; 3 Department of General Surgery, Saint Louis University Hospital, St. Louis, MO USA; 4 School of Medicine, St. Louis University, St. Louis, MO USA

**Keywords:** Appendectomy, Acute appendicitis, Missing data, Pediatric surgery, Missingness

## Abstract

**Background:**

Appendectomy is a leading pediatric surgical procedure, yet the impact of missing data handling on clinical conclusions remains unexplored in pediatric surgery. This study aimed to identify current reporting practices in the pediatric appendectomy literature and quantify how different missing-data strategies influence the identification of postoperative infectious-complication risk factors.

**Methods:**

A systematic review of pediatric appendectomy literature from 2023 was conducted across PubMed and Web of Science to assess missing data reporting. Subsequently, a retrospective analysis of the National Surgical Quality Improvement Program-Pediatric (NSQIP-P) database (2015–2022) was performed on 142,129 cases. Five strategies were compared: available case analysis, threshold-based exclusion, complete case analysis, simple imputation, and multiple imputation. Multivariable logistic regression models identified risk factors for five infectious outcomes, including combined infectious complications, organ space infections, deep incisional infections, superficial surgical site infections, and wound dehiscence.

**Results:**

The literature review identified 116 eligible articles, of which 54.3% failed to mention missing data. Only 7.3% used advanced statistical techniques, while 86.4% reported that a method relied on complete-case analysis. In the NSQIP-P cohort, 39.3% of records had missing values, primarily in height and race. While core predictors like American Society of Anesthesiologists (ASA) class and operative duration remained stable in multivariate logistic regression analysis, variables such as preoperative white blood cell count and anthropometric measurements fluctuated in significance depending on the handling method. Complete case analysis was the most conservative, identifying the fewest significant predictors.

**Conclusion:**

Methodological transparency regarding missing data is critically lacking in pediatric surgical research. Because the choice of data-handling strategy significantly alters the identification of clinical risk factors, researchers should prioritize multiple imputation over simple exclusion methods. Journals must mandate rigorous reporting of missingness to ensure the reliability of evidence-based surgical guidelines.

**Supplementary Information:**

The online version contains supplementary material available at https://doi.org/10.1007/s00464-026-13123-7.

Appendectomy remains one of the most frequently performed surgical procedures in the United States (US), particularly within the pediatric population. In fact, the ages 1–17 account for roughly 25% of all appendicitis cases, totaling approximately 75,000 cases each year [1], highlighting the procedure’s critical role in pediatric healthcare delivery.

To monitor and improve surgical outcomes, the American College of Surgeons established the National Surgical Quality Improvement Program (NSQIP) and its pediatric-specific counterpart, NSQIP-Pediatric (NSQIP-P). These programs are designed to collect rigorous perioperative data and track 30 day postoperative complications. NSQIP-P captures more than 145,000 cases annually from 150 hospitals across the US and six other countries [2]. This high-volume, multi-institutional database provides an essential foundation for clinical research and quality benchmarking.

Despite the depth of these databases, the integrity of clinical research often depends on how researchers address the inevitable challenge of missing values. Previous research has shown that methods for handling missing data can significantly alter study outcomes and conclusions. This phenomenon has been documented across various medical specialties, including orthopedics [3–5], oncological surgery [6], bariatric surgery [7], cardiac catheterization [8], thoracic surgery [9], post-operative complication surveillance [10], and cost-effectiveness studies [11].

However, to our knowledge, no such studies have been conducted to evaluate the impact of missing data handling in pediatric surgery. Given the high volume of procedures such as appendectomies, understanding how data gaps influence clinical conclusions is vital to evidence-based practice.

In this multi-phase study, we aimed to identify current practices for reporting missing values in the pediatric surgical literature and to demonstrate how different handling methods affect research outcomes. To achieve these goals, we conducted a comprehensive literature review on appendectomy and used NSQIP-P data to identify risk factors for post-appendectomy infectious complications. By applying various missing data handling techniques, we sought to quantify their influence on the identification of clinical risk factors and on the overall stability of surgical research findings.

## Materials and methods

The investigation was granted an Institutional Review Board exemption for non-human-subject research by Saint Louis University Medical School. This study utilized the Reporting of Studies Conducted using Strengthening the Reporting of Observational Studies in Epidemiology (STROBE) guideline [12].

### Review of appendectomy literature

To evaluate current practices in reporting missing data within pediatric appendectomy literature, we conducted a systematic review of articles published between January 1, 2023, and December 31, 2023. The literature search was conducted in PubMed and Web of Science to identify relevant peer-reviewed research.

Studies were eligible for inclusion if they were published in English, involved human subjects, and were available in full text. The scope was limited to the pediatric population, defined as children under 19 years of age. We included observational research, comparative studies, and clinical trials, provided they contained patient demographic analysis. To ensure the specificity of our findings, we excluded articles that reported on a mixture of appendectomy and other surgical procedures, focusing exclusively on studies dedicated to appendectomy outcomes.

The screening task was distributed among seven researchers (AH, MN, NK, GT, JQ, PR, and AT). To ensure rigor and minimize bias, each article was independently reviewed by two researchers in the group. When the two primary reviewers disagreed on a study’s eligibility, a third investigator was consulted to reach a final consensus and resolve the discrepancy.

### Missing data handling analysis

This retrospective analysis used NSQIP-P data from 2015 through 2022. Patients who underwent appendectomy were identified using Current Procedural Terminology (CPT) codes 44,950 (appendectomy), 44,960 (appendectomy for ruptured appendix with abscess or generalized peritonitis), 44,970 (laparoscopic appendectomy), and 44,979 (unlisted laparoscopic procedure of the appendix). To establish a unified study cohort, the NSQIP-P main participant use files were merged with the appendectomy procedure-specific files by case ID and year of operation across all study years. Predictor variables were selected based on established clinical relevance in existing literature [13–19]. Due to the low prevalence of certain clinical conditions within the pediatric population, specific comorbidities were consolidated into composite variables, as detailed in Supplemental Table 1. For the regression analysis, CPT codes were grouped by surgical approach: open (44,950 and 44,960) and laparoscopic (44,970 and 44,979). Similarly, American Society of Anesthesiologists (ASA) physical status classes 4 and 5 were merged due to low frequency, as were low-frequency racial categories.

**Table 1:** Prevalence of missing data across clinical variables, ordered by magnitude

Variables	No of missing values	% of missing values
Height (in)	34,261	24.106
Race	28,836	20.289
Preoperative WBC (K/mm^3^)	14,799	10.412
Pathology finding	5551	3.906
Operative finding of complicated appendicitis	5375	3.782
Nutritional support	5375	3.782
Ostomy*	5072	3.569
Weight (lbs)	370	0.260
ASA classification	232	0.163
Total operation time (min)	13	0.009
Childhood malignancy*	1	0.001
Wound classification	1	0.001

*No* number, *in* inch, *WBC* white blood cell, *lb* pound, *ASA* American Society of Anesthesiologists, *min* minutes

*Combined into combined comorbidities

The primary outcomes of interest were postoperative infectious complications, defined as superficial incisional, deep incisional, and organ space surgical site infections (SSIs), as well as wound dehiscence. In addition to individual analysis, a composite variable encompassing all four infectious complications was created for evaluation.

To account for the impact of missing data, laboratory values and comorbidities were assumed to be missing at random (MAR). Five distinct strategies were employed to address missingness:

- Group 1 (Available Case Analysis): Retention of the full cohort, performing analyses using all available data points of each variable.
- Group 2 (Threshold-Based Exclusion): Exclusion of patients with greater than 10% missingness, with the remaining data analyzed as available.
- Group 3 (Complete Case Analysis): Exclusion of any patient records containing missing observations.
- Group 4 (Simple Imputation): Application of mean imputation for numeric variables and mode imputation for categorical variables.
- Group 5 (Multiple Imputation): Creation of twenty distinct datasets through an iterative process, analyze them separately, and pool the results.

Univariate analysis was performed to identify differences between the complication and test groups, using independent *t*-tests for parametric continuous variables, Mann–Whitney *U* tests for nonparametric continuous variables, and chi-squared tests for categorical variables. Logistic regression models were subsequently fitted for the five infectious outcomes after excluding constant, duplicate, or collinear features. Measures of association were reported as odds ratios (OR) with 95% confidence intervals (CIs), with statistical significance defined by a *p*-value < 0.05 or CIs that did not cross 1.0. OR > 1 was identified as a risk factor, whereas OR < 1 was a protective factor. Primary statistical modeling was performed in R (PBC, Boston, MA), while preliminary descriptive statistics and data organization were conducted in Microsoft Excel (Microsoft Inc., Redmond, Washington).

## Results

### Review of appendectomy literature

**Fig. 1:**
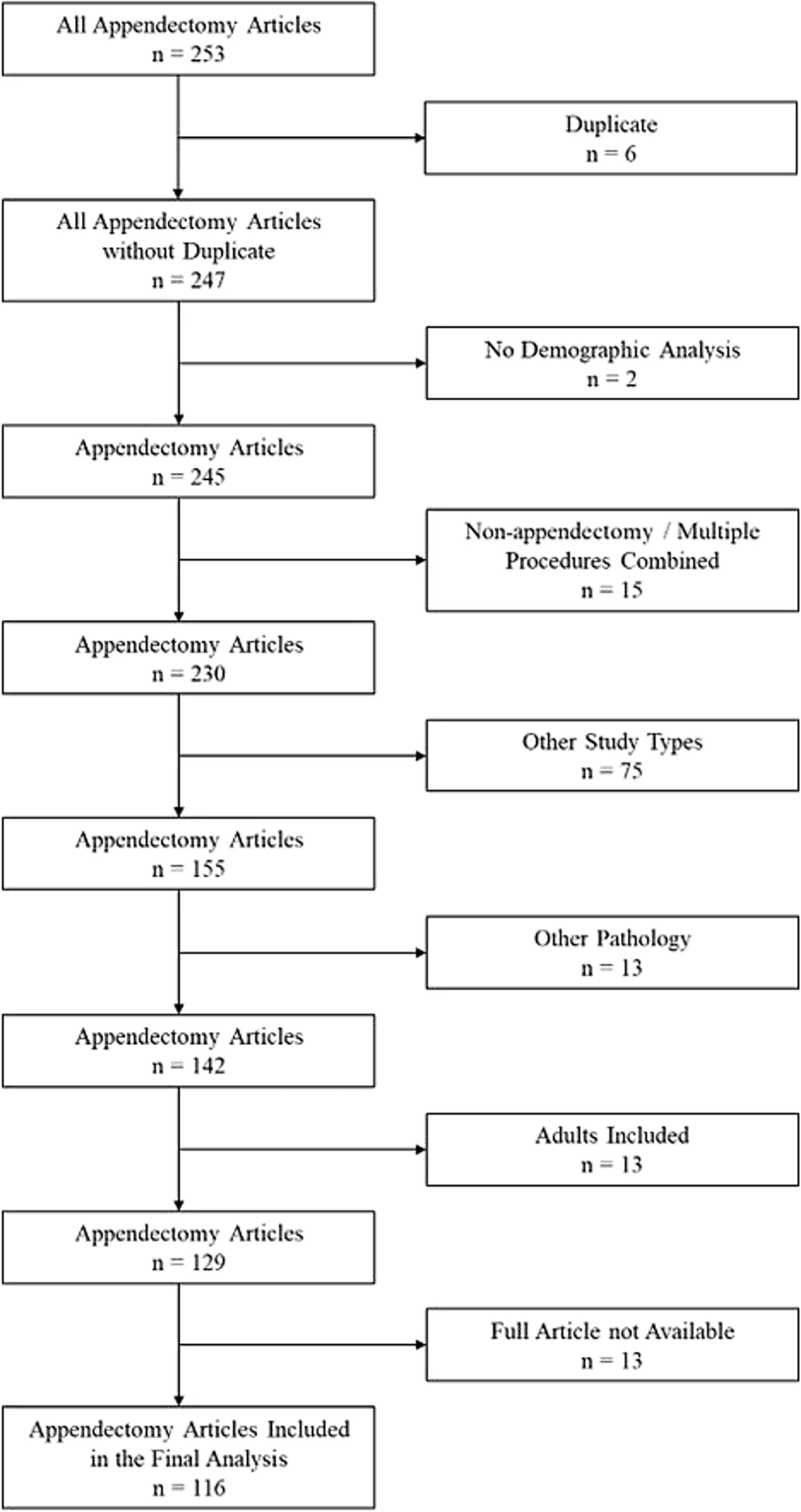
Flowchart of literature identification, screening, and inclusion for pediatric appendectomy

A total of 253 articles were identified through PubMed and Web of Science searches, of which 116 (45.8%) met the inclusion criteria (Fig. 1). The most frequent study design among the included literature was the cohort study (*n* = 79), followed by observational studies (*n* = 15). Both case–control studies and clinical trials were represented equally, with seven articles each.

Regarding the origin of the data used in these studies, the majority of researchers utilized local hospital data or datasets aggregated from multiple hospitals (*n* = 95). National databases were used in 18 studies, with NSQIP the most frequently cited national resource, appearing in eight of those studies.

**Fig. 2:**
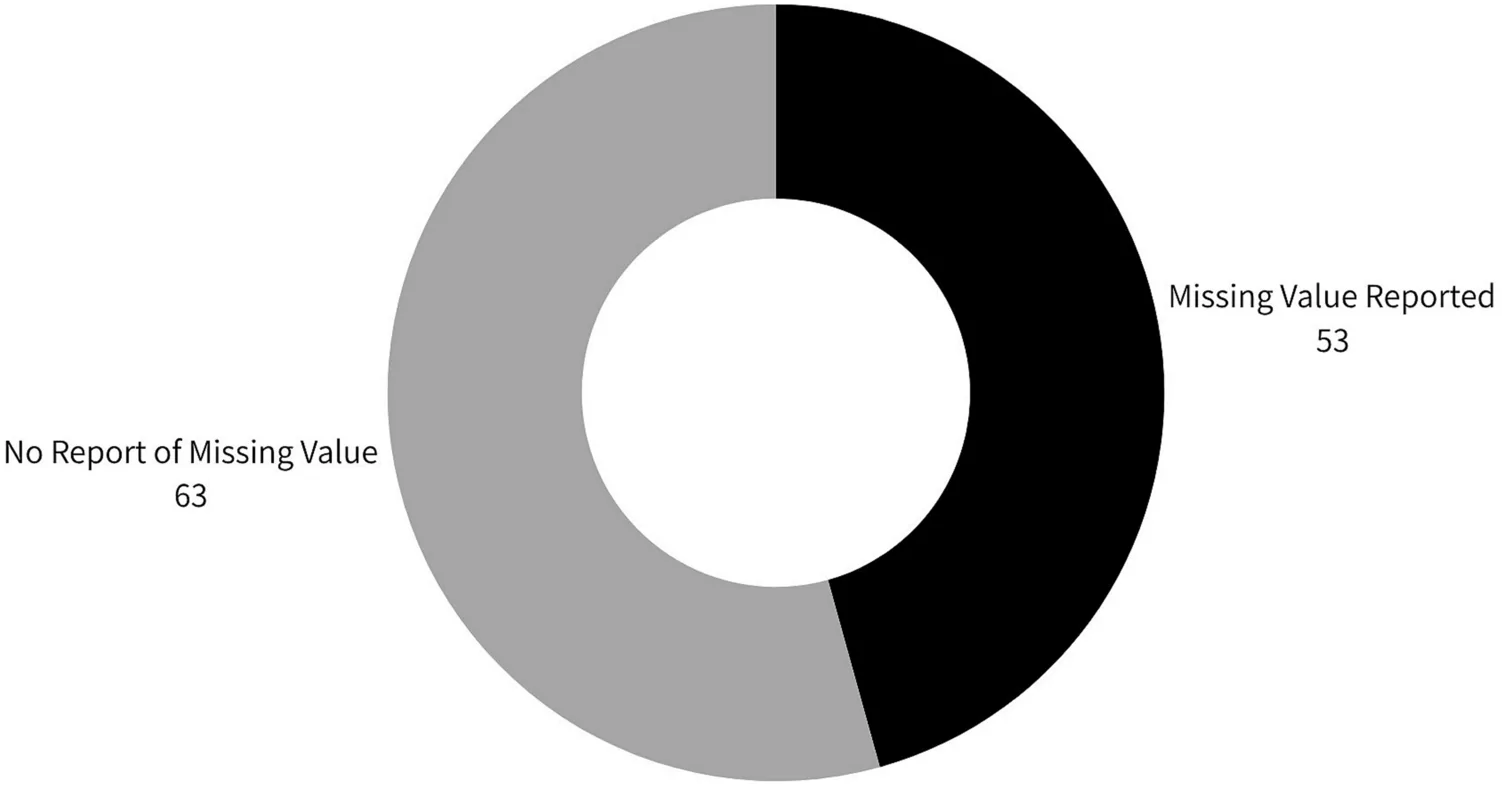
Proportion of academic articles addressing missing data in pediatric appendectomy research

**Fig. 3:**
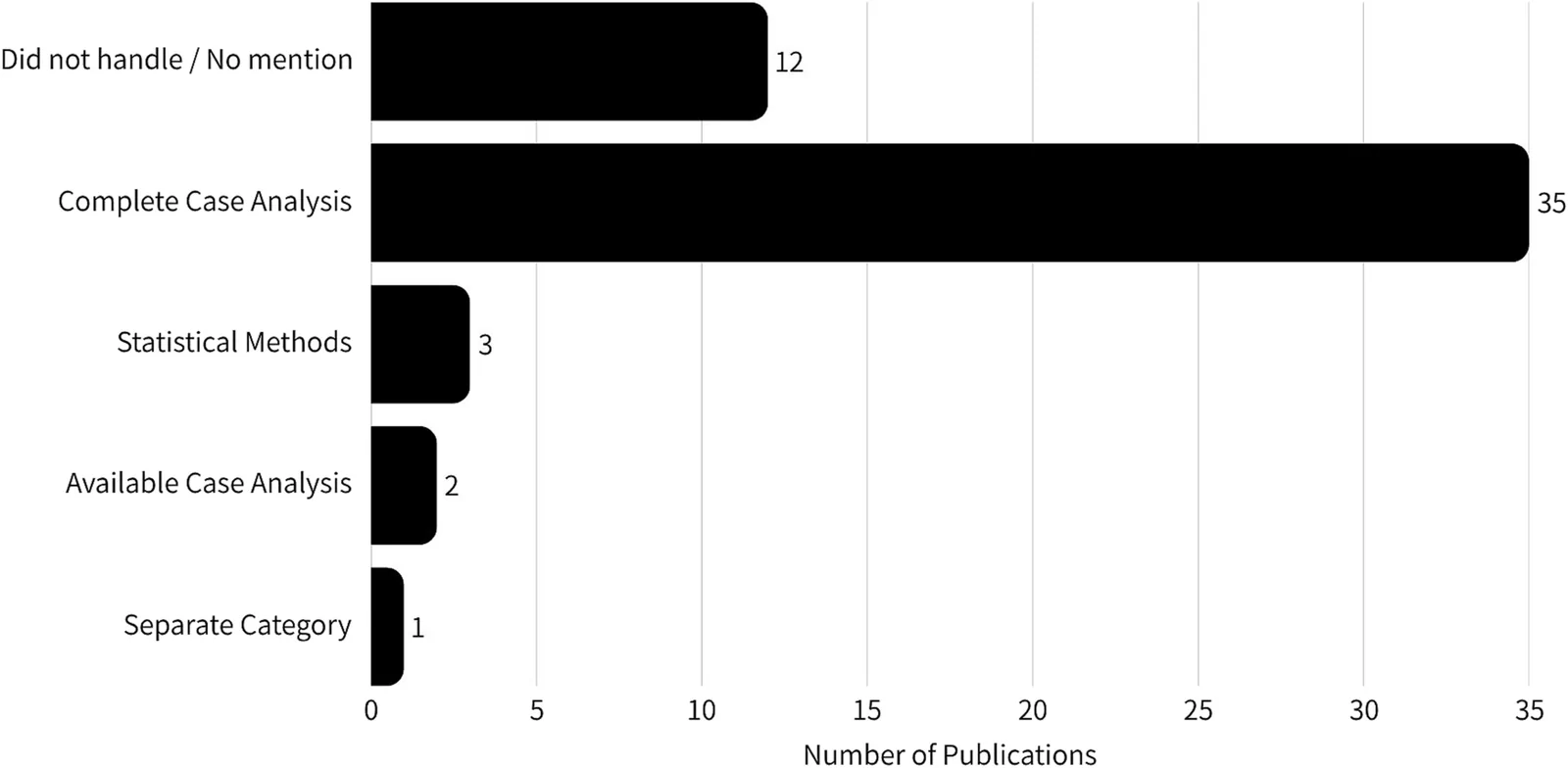
Distribution of missing value handling techniques across included studies

Analysis of data quality reporting revealed that 53 studies (45.7%) explicitly mentioned the presence of missing values (Fig. 2). However, only 41 of these articles described the methodology used to handle this missingness. The most common approach was to exclude cases with missing values from the analysis, i.e., complete-case analysis, a method adopted by 35 articles (86.4% of the studies that addressed missing data handling). In contrast, sophisticated statistical methods were used in only three studies (7.3%). Among these, one study employed mean imputation combined with sensitivity analysis [20], while the other two utilized multiple imputation [21, 22] (Fig. 3).

### Missing data handling

Of the 142,129 records initially identified meeting the inclusion criteria, 60.71% (*n* = 86,288) contained complete data across all study variables. An analysis of data completeness revealed that certain variables exhibited high rates of missingness, most notably height (24.11%) and race (20.29%) (Table 1).

The overall incidence of combined infectious complications was 4.12% (*n* = 5861), suggesting that the vast majority of the cohort did not experience infection-related adverse events. When stratified by specific complication type, organ-space SSI was the most prevalent, occurring in 2.99% of patients (*n* = 4244). This was followed by superficial SSI (1.02%, *n* = 1445), deep incisional SSI (0.12%, *n* = 172), and wound disruption (0.05%, *n* = 66) (Table 2).

**Table 2:** Comparison of outcome frequencies and cohort size across five strategies for missing value management

	G1	G2	G3	G4	G5
Overall population (*n*)	1,42,129	1,36,753	86,288	1,42,129	1,42,129
Overall infectious complications (%)	5861 (4.12)	5707 (4.17)	3834 (4.44)	5861 (4.12)	5861 (4.12)
Organ space SSI (%)	4244 (2.99)	4179 (3.06)	2883 (3.34)	4244 (2.99)	4244 (2.99)
Deep incisional SSI (%)	172 (0.12)	166 (0.12)	108 (0.13)	172 (0.12)	172 (0.12)
Superficial SSI (%)	1445 (1.02)	1363 (1.00)	843 (0.98)	1445 (1.02)	1445 (1.02)
Wound disruption (%)	66 (0.05)	65 (0.05)	43 (0.05)	66 (0.05)	66 (0.05)

*G1* group 1 (available case analysis), *G2* group 2 (threshold-based exclusion), *G3* group 3 (complete case analysis), *G4* group 4 (simple imputation), *G5* group 5 (multiple imputation)

The univariate analysis demonstrated that the statistical significance of variables for combined infectious complications and organ-space SSI remained consistent across the missingness handling strategies employed (Table 3 and Supplemental Tables 2 and 3). In contrast, the significance of several variables fluctuated in other complication categories. For deep incisional SSI, variables such as race, combined comorbidities, nutritional support, and preoperative sepsis showed varied levels of statistical significance across groups. Similarly, the significance of the surgical approach (CPT), ASA classification, intraoperative findings, and pathology findings for superficial incisional SSI switched depending on how missing data were managed. For wound disruption, nutritional support, and preoperative sepsis status, the primary variables demonstrated sensitivity to missingness handling (Supplemental Tables 4–6).

**Table 3:** Significance map: stability of univariate predictors for the overall infectious complications across missing data handling strategies

	G1	G2	G3	G4	G5	Interpretation
Demographics						
Age	< 0.001	< 0.001	< 0.001	< 0.001	< 0.001	Stable
Sex	< 0.001	< 0.001	< 0.001	< 0.001	< 0.001	Stable
Race	< 0.001	< 0.001	< 0.001	< 0.001	< 0.001	Stable
Clinical factors						
Combined comorbidities	< 0.001	< 0.001	0.005	< 0.001	< 0.001	Stable
Steroid use	0.2	0.2	0.13	0.2	0.2	Not significant
Preoperative sepsis	< 0.001	< 0.001	< 0.001	< 0.001	< 0.001	Stable
Preoperative WBC	< 0.001	< 0.001	< 0.001	< 0.001	< 0.001	Stable
Operative factors						
Wound classification	< 0.001	< 0.001	< 0.001	< 0.001	< 0.001	Stable
ASA classification	< 0.001	< 0.001	< 0.001	< 0.001	< 0.001	Stable
Total operation time	< 0.001	< 0.001	< 0.001	< 0.001	< 0.001	Stable
Complicated appendicitis	< 0.001	< 0.001	< 0.001	< 0.001	< 0.001	Stable

*G1* group 1 (available case analysis), *G2* group 2 (threshold-based exclusion), *G3* group 3 (complete case analysis), *G4* group 4 (simple imputation), *G5* group 5 (multiple imputation), *ASA* American Society of Anesthesiologists

In multivariable regression, the number of significant predictors across all five outcomes varied by missingness-handling strategy: 43 for Available Case Analysis (Group 1) and Threshold-Based Exclusion (Group 2), 36 for Complete-Case Analysis (Group 3), 46 for Simple Imputation (Group 4), and 40 for Multiple Imputation (Group 5) (Tables 4 and 5). Groups 1 and 2 produced identical sets of risk and protective factors across all outcomes, while Group 5 diverged from all other groups. Complete-case analysis yielded the most conservative results, and simple imputation identified the greatest number of significant predictors overall.

**Table 4:** Comparative logistic regression analysis: impact of missing value handling on predictors of postoperative complications (overall and the organ space surgical site infections)

	Overall infectious complications					Organ space SSI				
	G1	G2	G3	G4	G5	G1	G2	G3	G4	G5
Sex (Female—Ref.)										
Male	Risk	Risk	Risk	Risk	Risk	Risk	Risk	Risk	Risk	Risk
Race (White—Ref.)										
Asian	NS	NS	NS	NS	NS	NS	NS	NS	NS	NS
Black or African American	Risk	Risk	Risk	Risk	Risk	Risk	Risk	Risk	Risk	Risk
Other	Risk	Risk	Risk	Risk	NS	Risk	Risk	Risk	Risk	NS
Age (days)	Protective	Protective	Protective	Protective	NS	NS	NS	NS	NS	NS
Height (in)	NS	NS	NS	NS	Risk	Risk	Risk	Risk	NS	Risk
Weight (lbs)	Risk	Risk	NS	Risk	NS	NS	NS	NS	NS	NS
Combined comorbidities	NS	NS	NS	Risk	NS	NS	NS	NS	NS	NS
Preoperative steroid use	NS	NS	NS	NS	NS	NS	NS	NS	NS	NS
Preoperative nutritional support	Risk	Risk	Risk	Risk	Risk	NS	NS	NS	Risk	Risk
Preoperative sepsis	Risk	Risk	Risk	Risk	Risk	Risk	Risk	Risk	Risk	Risk
Preoperative inotropic support	NS	NS	NS	NS	NS	NS	NS	NS	NS	NS
Preoperative WBC	NS	NS	NS	NS	NS	Risk	Risk	NS	NS	NS
Surgical approach (Open—Ref.)										
Laparoscopy	NS	NS	NS	NS	NS	NS	NS	NS	NS	NS
Wound class (Clean—Ref.)										
Clean/contaminated	NS	NS	NS	NS	NS	NS	NS	NS	NS	NS
Contaminated	NS	NS	NS	NS	Protective	Protective	Protective	NS	Protective	Protective
Dirty/infected	Risk	Risk	Risk	Risk	Risk	Risk	Risk	Risk	Risk	Risk
ASA class (1—Ref.)										
2	Risk	Risk	Risk	Risk	Risk	Risk	Risk	Risk	Risk	Risk
3	Risk	Risk	Risk	Risk	Risk	Risk	Risk	Risk	Risk	Risk
4_5	Risk	Risk	Risk	Risk	Risk	Risk	Risk	Risk	Risk	Risk
Total operation time (min)	Risk	Risk	Risk	Risk	Risk	Risk	Risk	Risk	Risk	Risk
Op finding of complicated appendicitis	Risk	Risk	Risk	Risk	Risk	Risk	Risk	Risk	Risk	Risk
Pathology finding (Normal—Ref.)										
Appendicitis	NS	NS	NS	NS	NS	NS	NS	NS	NS	NS
Other appendiceal pathology	NS	NS	NS	NS	NS	NS	NS	NS	Risk	NS

*G1* group 1 (available case analysis), *G2* group 2 (threshold-based exclusion), *G3* group 3 (complete case analysis), *G4* group 4 (simple imputation), *G5* group 5 (multiple imputation), *Risk* risk factor, *Protective* protective factor, *Ref* reference, *in* inch, *WBC* white blood cell, *lb* pound, *ASA* American Society of Anesthesiologists, *min* minutes, *Op* operative

**Table 5:** Comparative logistic regression analysis: impact of missing value handling on predictors of postoperative complications (other infectious complications)

	Deep incisional SSI					Superficial SSI					Wound disruption				
	G1	G2	G3	G4	G5	G1	G2	G3	G4	G5	G1	G2	G3	G4	G5
Sex (Female—Ref.)															
Male	NS	NS	NS	NS	NS	NS	NS	NS	NS	NS	NS	NS	NS	NS	NS
Race (White—Ref.)															
Asian	NS	NS	NS	NS	NS	NS	NS	NS	NS	NS	NS	NS	NS	NS	NS
Black or African American	NS	NS	NS	NS	NS	Protective	Protective	Protective	Protective	NS	NS	NS	NS	NS	NS
Other	NS	NS	NS	NS	NS	NS	NS	NS	NS	NS	NS	NS	NS	NS	NS
Age (days)	Risk	Risk	Risk	NS	Risk	Protective	Protective	Protective	Protective	NS	NS	NS	NS	NS	NS
Height (in)	NS	NS	NS	NS	NS	Protective	Protective	Protective	Protective	NS	NS	NS	NS	NS	NS
Weight (lbs)	NS	NS	NS	NS	NS	Risk	Risk	Risk	Risk	NS	NS	NS	NS	NS	NS
Combined comorbidities	Protective	Protective	NS	NS	NS	Risk	Risk	NS	Risk	NS	NS	NS	NS	NS	NS
Preoperative steroid use	NS	NS	NS	NS	NS	NS	NS	NS	NS	NS	NS	NS	NS	NS	NS
Preoperative nutritional support	NS	NS	NS	NS	NS	NS	NS	NS	NS	Risk	Risk	Risk	NS	Risk	Risk
Preoperative sepsis	NS	NS	NS	NS	NS	NS	NS	NS	NS	NS	NS	NS	NS	NS	NS
Preoperative inotropic support	NS	NS	NS	NS	NS	NS	NS	NS	NS	NS	NS	NS	NS	NS	NS
Preoperative WBC	NS	NS	NS	NS	NS	NS	NS	NS	NS	NS	NS	NS	NS	NS	NS
Surgical approach (Open—Ref.)															
Laparoscopy	Risk	Risk	Risk	Risk	Risk	NS	NS	NS	Protective	Protective	Protective	Protective	Protective	Protective	Protective
Wound class (Clean—Ref.)															
Clean/Contaminated	NS	NS	NS	NS	NS	NS	NS	NS	Risk	NS	NS	NS	NS	NS	NS
Contaminated	NS	NS	NS	NS	NS	NS	NS	NS	NS	NS	NS	NS	NS	NS	NS
Dirty/infected	NS	NS	NS	NS	NS	NS	NS	NS	NS	NS	NS	NS	NS	NS	NS
ASA class (1—Ref.)															
2	NS	NS	NS	NS	NS	NS	NS	NS	NS	NS	NS	NS	NS	NS	NS
3	NS	NS	NS	NS	NS	NS	NS	NS	Risk	NS	NS	NS	NS	NS	NS
4_5	Protective	Protective	Protective	NS	NS	NS	NS	NS	NS	Risk	Risk	Risk	Risk	Risk	Risk
Total operation time (min)	Protective	Protective	Protective	Protective	Protective	Risk	Risk	Risk	Risk	Risk	Risk	Risk	Risk	Risk	Risk
Op finding of complicated appendicitis	Protective	Protective	Protective	Protective	Protective	Protective	Protective	NS	Protective	Risk	NS	NS	NS	Risk	Risk
Pathology finding (Normal—Ref.)															
Appendicitis	NS	NS	NS	NS	NS	NS	NS	NS	NS	Protective	NS	NS	NS	Protective	Protective
Other appendiceal pathology	NS	NS	NS	NS	NS	NS	NS	NS	NS	NS	NS	NS	NS	NS	NS

*G1* group 1 (available case analysis), *G2* group 2 (threshold-based exclusion), *G3* group 3 (complete case analysis), *G4* group 4 (simple imputation), *G5* group 5 (multiple imputation), *Risk* risk factor, *Protective* protective factor, *Ref* reference, *in* inch, *WBC* white blood cell, *lb* pound, *ASA* American Society of Anesthesiologists, *min* minutes, *Op* operative

For combined infectious complications, 8 variables were consistently identified across all five strategies: male sex, Race-Black, preoperative nutritional support, preoperative sepsis, dirty or infected wound class, ASA class, operative duration, and intraoperative findings of complicated appendicitis. Six additional variables—age, height, weight, combined comorbidities, Race-Other, and contaminated wound classification—were significant only under specific missingness treatments (Table 4). For organ-space SSI, the same 8 core predictors were identified, except for preoperative nutritional support, while 6 variables showed strategy-dependent significance (Table 4).

For deep incisional SSI, only 3 variables were consistently significant across all strategies—laparoscopic surgical approach, operative duration, and intraoperative findings of complicated appendicitis—all of which were protective, while age, combined comorbidities, and ASA class were inconsistent (Table 5). For superficial SSI, operative duration was the sole consistent predictor, with 11 additional variables reaching significance only under certain strategies (Table 5). For wound disruption, laparoscopic surgical approach (protective), ASA class 4–5, and operative duration were consistent across all models, while 3 variables—preoperative nutritional support, intraoperative findings of complicated appendicitis, and pathology findings—were strategy-dependent (Table 5).

## Discussion

To our knowledge, this study represents the first investigation into missingness reporting and the subsequent impact of missing-data handling on infectious complication risk factors in the pediatric appendectomy literature, using NSQIP-P and appendectomy-specific datasets. Our findings indicate that the vast majority of research in this field fails to address missing data or the methodologies used to manage it; notably, only 3 studies (7.3%) employed advanced statistical techniques.

The lack of transparency in managing missing data observed in our study reflects a broader systemic trend within medical literature. Previous reviews of hematological journals, for instance, revealed that 35% of articles failed to report missingness entirely, and among those that did, only 39% specified their handling methodology; notably, complete-case analysis was employed approximately 7 times more frequently than imputation techniques [23]. Similarly, research in intensive care medicine mirrors these deficiencies, with 36.4% of publications omitting missingness reporting and 45.5% of reporting articles relying on complete-case analysis, while only 4.5% utilized sophisticated statistical approaches such as MI [24]. Our findings align closely with these studies: 54% of the studies analyzed failed to report missing values, and among those that did, 81% addressed the issue through complete-case analysis. With only 7% of studies in our cohort applying advanced methodologies such as MI.

The second phase of our analysis demonstrated that the choice of missing-data handling significantly influences the identification of risk and protective factors for post-appendectomy infectious complications. Specifically, while univariate analyses of combined infectious complications and organ-space SSI, the most prevalent outcomes, yielded consistent results across all handling techniques, multivariate models revealed critical discrepancies. Complete Case Analysis (Group 3) identified the fewest significant predictors (36 total), suggesting that strict exclusion of incomplete records may diminish statistical power or introduce selection bias, potentially overlooking subtle but clinically relevant predictors. Simple Imputation (Group 4) identified the most significant predictors (46 total). In comparison, Multiple Imputation (Group 5) yielded an intermediate result (40 total), underscoring that imputation strategies are not interchangeable and can meaningfully alter model conclusions. Interestingly, the congruent results between Available Case Analysis (Group 1) and Threshold-Based Exclusion (Group 2), both identifying 43 significant predictors, suggest that in large datasets, removing variables with extreme missingness may not fundamentally alter model directionality, provided the remaining data remains representative. Conversely, the divergence observed between simple and multiple imputation models (Groups 4 and 5) highlights the complexity of addressing data gaps; while imputation aims to preserve sample size and mitigate bias, it can introduce new variables into the significance pool, necessitating a cautious balance between maximizing data utility and maintaining the integrity of the original observations [11].

Previous studies in adult orthopedic and cancer surgery have indicated that preoperative laboratory data are among the most frequently missing variables, especially among younger cohorts [3, 4, 6]. In this study, we focused specifically on the white blood cell (WBC) count, a primary diagnostic component in acute appendicitis, and observed a missingness rate of 10.4%, which is relatively low compared to similar large-scale registry analyses. Despite this relatively high degree of data completeness, the WBC count functioned as an unstable risk factor for organ space SSI, achieving statistical significance only within the Available Case Analysis and Threshold-Based Exclusion frameworks (Groups 1 and 2, respectively). This variability suggests that the perceived clinical importance of a laboratory marker may vary depending on how missingness is handled.

### Postoperative infectious complications

As appendectomy remains one of the most frequently performed procedures in the pediatric population, a substantial body of literature has been dedicated to investigating associated postoperative complications. Research indicates that both extremes of age are correlated with elevated risks; specifically, infants and preschool-aged children often experience rapid disease progression and higher complication rates, while older age is linked to a higher incidence of postoperative sepsis [13, 14]. In the current logistic regression analysis, older age was identified as a common protective factor for overall infectious complications and superficial SSI, a variable protective factor for organ space SSI and wound disruption, and a variable risk factor for deep incisional SSI. However, these effects were consistently marginal—typically less than 1%—suggesting they may not reach the threshold of clinical significance (Supplemental Tables 7–11).

Furthermore, longer operative duration (OT) is independently associated with increased morbidity [16, 19]. Prior evidence suggests that for every one-minute increase in OT, the likelihood of developing any SSI, superficial SSI, or organ-space SSI increases, with a 15 min increase predicting a 1.16-fold increase in the probability of these outcomes [16]. In this study, total OT was a common risk factor for overall infectious complications, organ space SSI, superficial SSI, and wound disruption, while serving as a common protective factor for deep incisional SSI. Consistent with other demographic variables, the effects were small and often stayed below 1% (Supplemental Tables 7–11).

Finally, while laparoscopic appendectomy is generally associated with lower SSI rates than open surgery [14, 25, 26], our study revealed a more complex relationship. The laparoscopic approach was identified as a common risk factor for deep incisional SSI, yet it served as a robust protective factor for wound disruption and a variable protective factor for superficial SSI. Notably, for deep incisional SSI, the odds ratio was greater than 3.0 across all missingness-handling techniques, while for wound disruption, the odds ratios ranged from 0.16 to 0.2, indicating substantial clinical significance (Supplemental Tables 9–11). It is important to acknowledge, however, that over 94% of cases in this cohort were performed laparoscopically; this significant procedural imbalance may have influenced the logistic regression results.

### Missing data and its handling

To contextualize the statistical implications of our results, it is essential to consider the three primary mechanisms of data missingness: missing completely at random (MCAR), missing at random (MAR), and missing not at random (MNAR). MCAR occurs when the probability of a value being missing is entirely independent of both observed and unobserved characteristics within the study sample, often resulting from stochastic errors such as administrative data entry mistakes or lost files [27, 28]. While simple techniques like complete-case analysis remain unbiased under MCAR conditions, they are inherently limited by a notable loss of statistical power [27, 29].

In contrast, MAR arises when the probability of missingness depends on observed data points but not on the unobserved values themselves [27, 28, 30]. A typical example involves missing weight data for younger patients who may utilize healthcare facilities less frequently than older cohorts; once age is controlled for, the missingness is considered independent of the actual unobserved weight [30]. Because MAR is a requisite assumption for sophisticated statistical approaches such as MI, it served as the primary assumption in our analysis [27]. Finally, MNAR exists when the probability of missingness is tied to unobserved information or the missing value itself, such as severely depressed patients being unable to attend follow-up assessments due to the severity of their symptoms [27, 28, 31]. Since the information required to identify MNAR is, by definition, absent from the dataset, it is impossible to distinguish MAR from MNAR using observed data alone [31]. Consequently, standard analytical methods in MNAR scenarios often produce biased estimates, as the underlying driver of missingness is fundamentally linked to the outcome of interest [24, 27–31].

As noted above, the complete case analysis introduces systematic bias unless the data satisfy the stringent MCAR requirement. Nevertheless, current publications in the field continue to rely primarily on complete-case or available-case analysis, often overlooking the potential for biased results. This practice persists despite a broad consensus in medical statistics literature recommending MI as a more robust framework for addressing missingness while maintaining the integrity of the original sample [29–33].

While the ongoing evolution of artificial intelligence and deep neural networks may soon provide even more advanced alternatives to traditional MI, the immediate priority remains methodological transparency [34, 35]. Therefore, it is recommended that academic journals mandate the comprehensive reporting of missing data proportions, along with the specific strategies used to address them, thereby enabling a more critical appraisal of the validity of study conclusions.

### Limitations

Several limitations of this study warrant consideration. First, the literature review was constrained by a relatively narrow temporal window and focused primarily on major indexing systems; consequently, small-scale investigations, articles published outside of the search period, or international studies published outside these databases may have been overlooked. Second, our analysis assumed that missing values were missing at random. However, the precise nature of missingness in the NSQIP-P database remains difficult to verify, and the presence of data missing not at random could affect the validity of our findings. Furthermore, this study is subject to the inherent limitations of large national registries, including potential inconsistencies in data entry and a 30 day follow-up window that may be insufficient to capture all delayed infectious complications. Finally, while we utilized several techniques for handling missing data, our objective was not to provide a comprehensive comparison of all available statistical strategies, but rather to demonstrate how the choice of methodology can significantly alter the identification of clinical risk factors.

## Conclusions

In conclusion, this study reveals a critical lack of methodological transparency in pediatric appendectomy research, with nearly half of the existing literature failing to report missingness and only 7.3% using advanced statistical handling techniques. Our analysis of the NSQIP-P database demonstrates that the choice of missing-data management is a fundamental determinant of study results. At the same time, core predictors such as ASA classification and operative duration remain statistically significant; many other variables, such as preoperative laboratory values and anthropometric measurements, show high sensitivity to the chosen analytical framework.

The conservative nature of complete-case analysis often reduces statistical power, potentially obscuring clinically relevant predictors and introducing selection bias. To ensure the development of reliable risk-stratification tools and evidence-based clinical guidelines, it is imperative that researchers adopt sophisticated imputation methods and that academic journals mandate transparent reporting of missing data handling to distinguish genuine clinical trends from statistical artifacts.

## Supplementary Information

Below is the link to the electronic supplementary material.

Supplementary file1 (XLSX 96 KB)
